# Measuring Mental Wellbeing of Children via Human-Robot Interaction: Challenges and Opportunities

**DOI:** 10.1075/is.21027.abb

**Published:** 2023-03-24

**Authors:** Nida Itrat Abbasi, Micol Spitale, Peter B. Jones, Hatice Gunes

**Affiliations:** 1 Department of Computer Science and Technology, University of Cambridge (15 JJ Thomson Ave, Cambridge CB3 0FD); 2 Department of Computer Science and Technology, University of Cambridge (15 JJ Thomson Ave, Cambridge CB3 0FD); 3 Dept. of Psychiatry, University of Cambridge (Young Peoples Centre Douglas House, 18b Trumpington Rd, Cambridge CB2 8AH); 4 Dept. of Computer Science and Technology, University of Cambridge (15 JJ Thomson Ave, Cambridge CB3 0FD)

## Abstract

During the last decade, children have shown an increasing need for mental wellbeing interventions due to their anxiety and depression issues, which the COVID-19 pandemic has exacerbated. Socially Assistive Robotics have been shown to have a great potential to support children with mental wellbeing-related issues. However, understanding how robots can be used to aid the measurement of these issues is still an open challenge. This paper presents a narrative review of child-robot interaction (cHRI) papers (IEEE ROMAN proceedings from 2016-2021 and keyword-based article search using Google Scholar) to investigate the open challenges and potential knowledge gaps in the evaluation of mental wellbeing or the assessment of factors affecting mental wellbeing in children. We exploited the SPIDER framework to search for the key elements for the inclusion of relevant studies. Findings from this work (10 screened papers in total) investigate the challenges in cHRI studies about mental wellbeing by categorising the current research in terms of robot-related factors (robot autonomy and type of robot), protocol-related factors (experiment purpose, tasks, participants and user sensing) and data related factors (analysis and findings). The main contribution of this work is to highlight the potential opportunities for cHRI researchers to carry out measurements concerning children’s mental wellbeing.

## 1 Introduction

Mental wellbeing is an integral part of humans’ overall health and is often referred to as subjective wellbeing (i.e., our perception and evaluation of our life)^1^. During the last decade (as reported by the Mental Health in Children and Young People (MHCYP) survey in the United Kingdom^2^ and the Centers for Disease Control (CDC) in the United States^*^), an increasing number of mental wellbeing related issues in children has emerged. These mental issues have been amplified further during the pandemic by increased isolation due to school closures, limited finances, academic pressures and in general physical distancing from peers and friends^3, 4^. Mental wellbeing concerns in children could also lead to other problems related to stress management and negative academic performance. Psychological interventions can help children overcome these problems and promote mental health and wellbeing in children^5^. However, children are very reluctant to disclose sensitive information and it often takes months of dedicated trust building for therapists to get the children to open up^6–9^.

Socially Assistive Robotics (SARs) is an engaging and effective tool for supporting people with special needs (e.g., children with autism^10^, elderly^11^) in general, and children experiencing mental wellbeing related issues in particular^12^. SARs can provide a useful addition to traditional methods (e.g., therapy with a human professional), as robot behaviour can be manipulated to cater to individual needs. However, it is important to note that SARs should be used as a tool (not a substitute) to aid human therapists and clinical professionals as their expertise far exceed the capabilities of the robots. Past work^13^ has demonstrated that children are more likely to disclose confidential information to robots than adults. Having a robot as an interviewer can be very helpful, as the otherwise daunting scenario of a psychological setting can be made less intimidating with the presence of an agent that is child-like and appears as a peer^13, 14^.

This paper aims to investigate the current state of the art in cHRI research on mental wellbeing to understand potential research gaps and possible future directions of robot applications to aid measurements of mental health. Toward this goal, we have conducted a narrative review of empirical studies in cHRI research exploiting the SPIDER framework^15^ to search, in a rigorous way, the key elements of the studies to include. Our search procedure consisted of two steps: (1) reviewing the titles and the abstracts of the proceedings of IEEE RO-MAN in the last 5 years (2016-2021). We selected IEEE RO-MAN since the conference provides a pioneering forum for cHRI research by accepting contributions from multidisciplinary fields: computer science, artificial intelligence, psychology, cognitive sciences, robot technology, and ethics, and (2) keyword-based manual search of research articles on Google Scholar from the last 15 years. This search resulted in journal articles (from the International Journal of Social Robotics, Frontiers of Robotics and AI, Interaction Studies and Procedia Engineering) and conference articles (International Conference on Ubiquitous Robots and Ambient Intelligence and International Conference on Intelligent Robots and Systems).

Our main research question was the following:

*“What are the challenges that cHRI researchers face while conducting mental wellbeing related studies, and what are the opportunities for future research?”*.

As a result of our narrative review, we have also discussed potential opportunities for cHRI researchers to consider while designing studies about mental wellbeing in children.

The contributions of this paper are the following:

1. A discussion of the state of the art in current cHRI research about mental wellbeing;
2. The identification of the most relevant challenges faced by cHRI researchers in the field of mental wellbeing;
3. The investigation of research gaps and opportunities for cHRI researchers for designing mental wellbeing related studies.

The rest of the paper is structured as follows: Section 2 summarizes the background and related works that informed this research, Section 3 describes the methodology for running the narrative review of cHRI for mental wellbeing, Section 4 reports the results obtained while conducting this review, Section 5 lists the main challenges of conducting cHRI studies, Section 6 discusses the opportunities in cHRI research, and Section 7 concludes the paper and proposes directions for future research.

## 2 Background

In the following sections, we have reported relevant works about current standardised mental wellbeing assess-ments in children and the applications of Socially Assistive Robots (SARs).

### 2.1 Measurements of Mental Wellbeing in Children

An early assessment of children’s mental wellbeing is extremely important for their lives because it helps prevent mental issues that can impact their development, self-esteem, and academic performance. For instance, a study by Boo et al.^16^ found that psychological distress affects mindfulness in young adults, significantly disturbing their academic performance. Current methods to measure children’s mental wellbeing are based on standardized tests^17^, which have many limitations.

First, most of these tests are very long self-report questionnaires that often lack attractiveness and engagement, which could demotivate children^18^. Second, these tests are not always effective in identifying the markers of underlying psychological distress that are often not noticed until very late. This is mainly due to the fact that the tests are not appropriate for children of all ages. For example, self-report tests are not always suitable for very young kids who may have difficulties understanding some of the statements in the test, and therefore they may not result in valid and reliable data^19^. Finally, even children in regular contact with counselling services might be restricted in confiding in another person (therapist or teacher) who they might perceive as an authority figure^14, 20^.

Interactive technologies have been shown to have great potential to support mental wellbeing measurements, especially in children^21^. Specifically, robots, smart toys, and other conversational agents have shown to be effective in overcoming the above-mentioned limitations. In fact, these technologies are very engaging for children who desire to interact with them even for long periods^18^ and children from an early age have shown interest in such technologies^22^. Williams et al.^23^ have demonstrated that smart toys have been useful in gaining children’s trust and influencing their perspectives on certain transgressions. To this end, robots and smart toys can help identify mental wellbeing issues without adopting paper-based tests but by using conversational approaches^24^. However, in the case of smart toys, age is an important aspect that should be considered when designing studies related to wellbeing. For example, older children (around 10 years and above) might consider disclosing to a smart toy, like a doll, as juvenile and might not be able to take the experience with the required level of seriousness^25, 26^. Thus, using robot-based measurements in such cases might be beneficial as robots could help children to be more candid about their mental wellbeing.

### 2.2 SARs and cHRI

During recent decades, previous works^18, 27, 28^ have explored the use of SARs to support children in educational, therapeutic, rehabilitation, and edutainment contexts. For example, the Adaptive Strategies for sustainable long-term interaction (ALIZ-E) project started in 2010 to investigate the interaction of children and adolescents and small robots over a longer duration, lasting from a few hours to even a couple of days^29, 30^. The project aimed to construct long-term interaction between robots and children so they could be successfully employed as tools in education or even companionship. It offered a coordinated multimodal perspective to address common challenges in HRI, using repeated but well-defined experimental scenarios where the robot interacted with children in varying conditions^30^. The target population for the ALIZ-E project were 7-11 year old diabetic children. The project’s main objective was to develop robot companions that interacted with children and help them better manage their diabetes^31, 32^. These companion robots have also been employed in patient settings where they have made hospital visits less intimidating^32^. The activities performed during the interaction sessions formed the basis for evaluating individual modules in the wild^33–35^. The experimental tasks were designed to complement the robot’s role as a mentor for better management of the health condition of the children^31, 32^. These included dancing^36^ and imitation games (physical activities for weight management)^30^, quiz and math games (non-physical interaction primarily using language^)30,37^ and Sandtray game (promoting awareness regarding their dietary necessities specific to their medical condition^)29,30^. The primary objective of these activities was to foster smooth social interaction patterns between the robot companion and the child, and did not focus on task performance or even completion of the activity^29^. Another relevant project in the field of cHRI was the Development of Robot-Enhanced therapy for children with Autism spectrum disorders (DREAM). This project aimed to develop advanced social robots that can function autonomously by decoding cues from the movement and emotion of the child, to provide robot therapy, aided by minimalistic instructions from the therapist^38^. The project aimed at the development of child-specific robotic interaction where the therapist modulated the robot behaviour to cater to the specific behavioural traits of the child with ASD^38–40^.

Other notable applications of cHRI include edutainment robotics, where robots have been used as tutors and teaching aids^41, 42^. Several studies have employed robots in schools where children have shown high engagement while it performed various academic and non-academic activities^42–44^. In a study conducted using primary school children, Baxter et al.^45, 46^ demonstrated that using robots in academics has improved classroom performance and engagement in children. It was noted that personalised interaction provided more engagement and better acceptance than non-personalised interaction. However, learning overall had been improved irrespective of the interaction methodology.

Many studies have shown that the increased acceptance of using robots in education has been consistent over all age groups of children^44, 47^. Venture et al.^47^ demonstrated high participation of 3-5 years old when a robot was employed in the nursery school. Studies have also shown that older children (above 12 years) have demonstrated high acceptability when robots have been used in their classrooms as teaching aids^48^. Even across different cultures, the responses of children have been positive with some variations (for example, expressivity) in their behaviour while interacting with the robot^49–51^.

There have been the following notable instances in which robots have been used to conduct interview-based assessments in children. Firstly, Bethel et al.^52^ have employed robots to identify cases concerning children’s bullying. Secondly, Godoi et al.^14^ have proposed using robots to conduct interviews regarding violence and abuse in children. Though Bethel et al.^52^ have only found measurable differences in one component (disclosing teasing incidences occurring to other classmates) of their interview between human and robotic interviewers, it does warrant future works to investigate if robots can be used to extract sensitive information from children. As such, employing robots for psychological screening, especially regarding mental wellbeing in children, still remains largely unexplored. This could be because robots being used for carrying out psychological measurements appear to be unfeasible as therapists and coaches require a significant duration of relationship building to gain the participant’s trust^53, 54^. However, robot-based evaluations can be very beneficial, originating from the fact that several online and e-mental health programmes have been successful with adults suffering from anxiety^55^, depression^56^ and alcohol consumption^57^. Having a psychologist or a coach modulate these intervention programs seem to achieve better results, but the self-motivated initiatives have also produced favourable outcomes^23, 58^. Children can also be more comfortable disclosing information to an agent^13, 14, 59^ whom they perceive will have some level of confidentiality as compared with a human counterpart who they might consider intimidating and ‘in-charge’^20, 60, 61^. Thus, using SAR could potentially be a solution for psychological measurements in children.

## 3 Methodology

We conducted a narrative review of the state of the art in robot-based measurements for children’s mental wellbeing and factors that might affect child mental wellbeing (e.g., social status^62^, temperament^63^, engagement^64^, mood^4^, personality^65^, interaction with other humans^64^, emotion^66^, mental state^67^ and concentration^68^). The psychological variables measured above can be related to either the components of subjective wellbeing mentioned in^1^ or the realms of wellbeing defined in^69^. Due to the lack of papers that focused solely on robotised evaluation of mental wellbeing of children, we have broadened our search to papers that measured robotised assessments of psychological factors that affect mental wellbeing in children.

To address our research question about the challenges that cHRI researchers face while conducting mental wellbeing related studies and the opportunities for future research, we have explored the following sub-questions:

1. **RQ1:** What is the main purpose of the study?
2. **RQ2:** What are the age-groups of children considered as participants?
3. **RQ3:** Are the studies task-based or do they encourage free-flow interaction between the robot and the participants?
4. **RQ4:** What are the robotic platforms used?
5. **RQ5:** What is the degree of autonomy of the robots used?
6. **RQ6:** What are the user sensing measures that have been used and evaluated in the study?
7. **RQ7:** What data analysis methodologies have been employed?
8. **RQ8:** What are the key findings in these studies?

To this end, we exploited the SPIDER framework^15^ searching in a rigorous way the key elements of studies to include. We selected a body of experimental works representative of the domain and extracted and analysed qualitative data from the papers included in this study.

### 3.1 Term Definitions

We defined the terms we considered relevant for this paper in the following list. However, many terms may have different meaning depending on the application context.

- *Mental Wellbeing:* Mental wellbeing is defined as subjective wellbeing that “consists of three interrelated components: life satisfaction, pleasant affect, and unpleasant affect. Affect refers to pleasant and unpleasant moods and emotions, whereas life satisfaction refers to a cognitive sense of satisfaction with life"^69–71^.
- *Mental Health:* According to the World Health Organisation (WHO), mental health is “a state of wellbeing in which every individual realises his or her own potential, can cope with the normal stresses of life, can work productively and fruitfully, and is able to make a contribution to her or his community"^72^.
- *Psychological Measurements:* Psychological measurement focuses on developing, testing, and validating tools to measure psychological qualities (e.g., emotions, cognitions, and personality).
- *Robotised Measurements:* We defined robotised measurements as the assessment of standardised variables that have been developed and tested via robots.
- *Socially Assistive Robots:* SARs have the goal “to create a close and an effective interaction with a human user for the purpose of giving assistance and achieving measurable progress in convalescence, rehabilitation and learning"^73^. SARs have been shown to be an engaging and effective tool to support people with special needs (e.g., children with autism^10^ and the elderly^11^).

### 3.2 Search Strategy

For our narrative review, we followed a two-step search strategy:

1. We condensed our research over the last 5 years (from January 2016 to January 2021). The search for relevant papers was run according to the SPIDER format^15^ as depicted in Table 1 and using the following keywords (synonyms and tenses): "robot^*^", "robotised", "robotic^*^", "assessment", "measurements", "estimation", "evaluation", "mental", "child" and "children". This search query has been searched within the IEEE ROMAN proceedings because they collect emerging works in the field of cHRI.
2. In order to conduct a more thorough search, the research team manually searched the same keywords on Google Scholar, adding relevant works that were excluded from the database search. For the keyword searching procedure on Google Scholar, the year of publication was expanded to the last 15 years (2006-2021) to produce a more comprehensive search for relevant publications. This results in papers from venues other than IEEE RO-MAN, including journal articles from the International Journal of Social Robotics, Frontiers of Robotics and AI, Interaction Studies, and Procedia Engineering, and conference articles from the International Conference on Ubiquitous Robots and Ambient Intelligence and International Conference on Intelligent Robots and Systems.

Studies about mental wellbeing were identified, and a research team member read through the titles and the abstracts to determine their suitability for inclusion in this work.

### 3.3 Selection of Relevant Publications

Our research team first selected the relevant works based on a set of inclusion and exclusion criteria, screening them from titles and keywords.

We decided to include papers that:

- involve children;
- address the assessment (i.e., measurements) of mental wellbeing or factors relating to mental wellbeing;
- present an empirical study;
- use a robot for the study interaction;
- contain in their title or keywords the search terms described in Section 3.2.

On the other hand, we excluded papers that:

- did not contain the combination of keywords described above in their title.
- are not in English;
- are surveys or literature reviews;
- are inaccessible to the research team.

Finally, one research team member read all of the relevant research articles identified through the title and keyword screening process to determine their eligibility.

### 3.4 Data Extraction and Analysis

In order to extract the data to address our research questions, we identified the main variables that correspond to the challenges described in the screened papers. We read through the discussion, conclusions, limitation, and future works sections of each study, and then we ran a data-driven thematic analysis^74^ to extract the most relevant theme patterns that correspond to the main challenges of each study. We extracted the qualitative data from the papers included in this study, identifying patterns (i.e., challenges and research gaps). Finally, we analyzed the data that was presented, organized by patterns in Section 4.

## 4 Results

**Figure 1:**
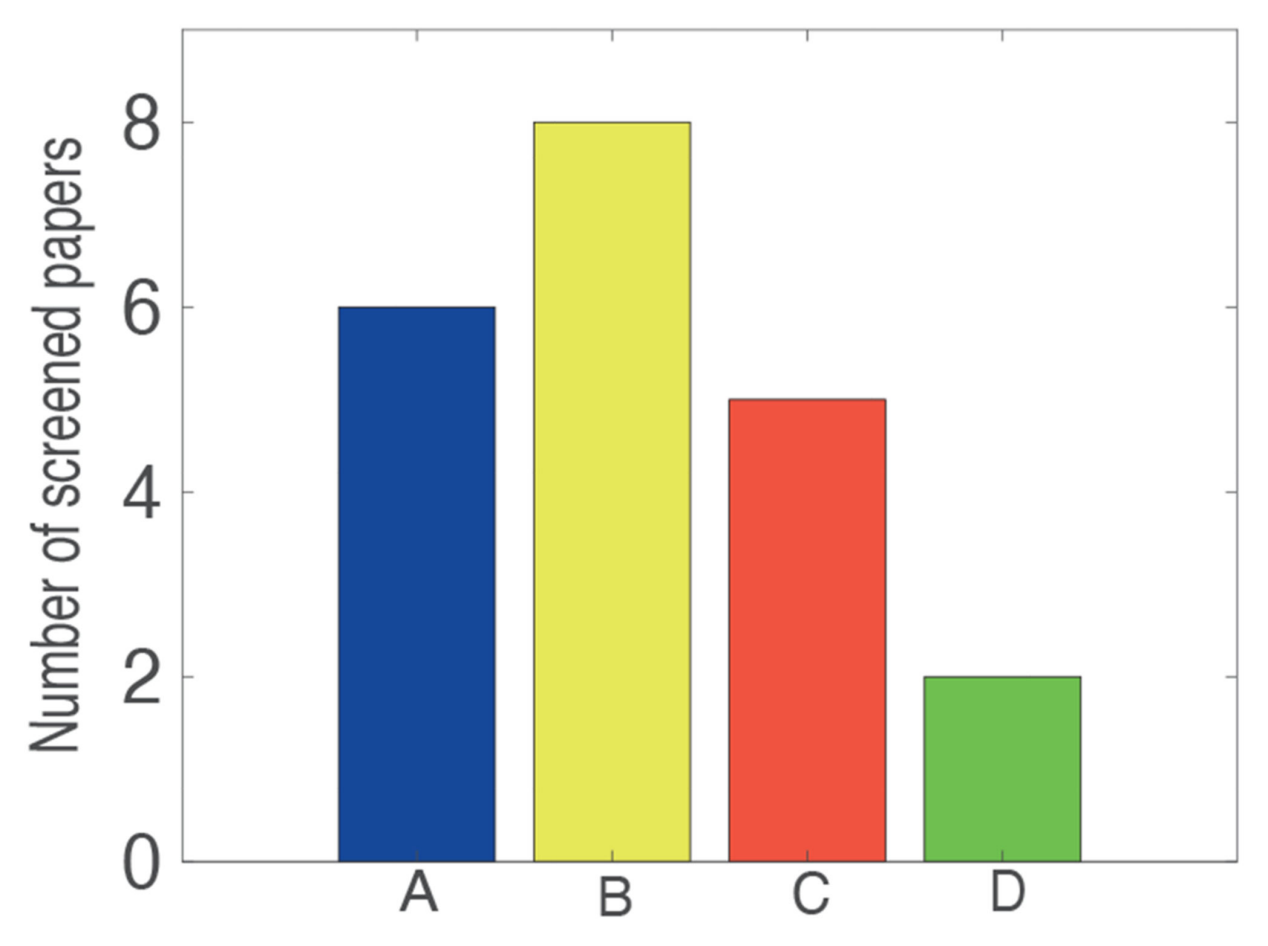
Four challenges have been predominantly identified in the screened papers: (A) Functionality of the robotised setup, (B) Protocol related limitations, (C) Establishing less constrained/more naturalistic and prolonged interactions and (D) Development of a validation protocol.

We included 10 studies (Table 2) in our narrative review. The following sections discuss four main challenges that have been identified by reviewing the screened cHRI studies (see Figure 1) as follows: 1) functionality of the robotised setup; 2) protocol-related limitations, 3) establishing less constrained/more naturalistic and prolonged interactions, and 4) development of a validation protocol. We have identified these challenges by reading the discussions, conclusions, limitations and future work sections of the screened manuscripts. For each challenge, we have attempted to answer our 8 sub-research questions (reported in Section 3). Note that some of the screened studies have highlighted multiple challenges, and so they have been repeated throughout the following sections (see Table 2).

### 4.1 Functionality of the Robotised Setup

One of the main challenges that emerged from our analysis was the functionality of the robotised setup since the capabilities of the robot are limited by its sensing abilities (usually restricted to only audio-visual sensing) or the estimation algorithms (limitations in performance/accuracy), restricting its ability to perform accurate evaluation of user behaviour. In addition, due to computing power constraints, most robotic platforms have not been designed for prolonged interactions. For instance, the robotic platform named Nao developed by Softbank robotics^†^ cannot be used for extended periods due to the amount of heat generated during its functioning, that could damage the robotic platform itself ^‡^.

**Figure 2:**
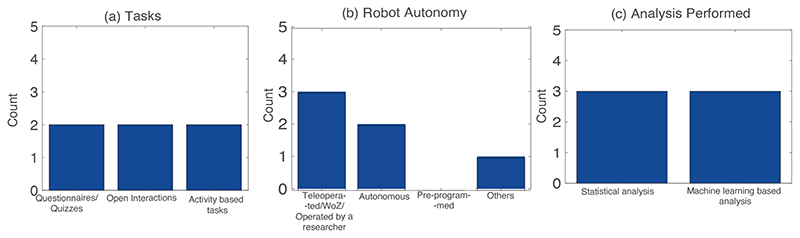
Bar graphs showing the quantitative summary of the results for the ‘Functionality of the Robotised Setup’ challenge: (a) Tasks performed in these studies (RQ3), (b) Robot autonomy in the studies screened (RQ5), and (c) Analysis performed in the above studies (RQ7).

Figure 2 and Table 3 contain the results from studies that have mentioned ‘functionality of the robotised setup’ as one of their challenges/future works. We have observed that 6 out of 10 studies mentioned that this limitation posits a challenge in accurately carrying out cHRI studies, and some of these studies have suggested ways in which they intend to overcome this limitation in their future works (Table 3 and Figure 1). Bethel et al.^52^ have mentioned that their future works would include expansion of the robot’s usage of sensors for precise measurements and superior autonomy while conducting the interviews. Kanda et al.^80^ have mentioned that better user sensing technology is required for more accurate autonomous interaction. Sano et al.^76^ have mentioned that precise cHRI studies require better algorithms that can augment the robot’s functionality. Rudovic et al.^77^ have suggested that the capabilities of the robotised setup need to be supplemented so that the interactions can be scaled up to augment the work performed by subject matter experts. Komatsubara et al.^75^ have suggested adding environment-based sensing to improve tracking and identification during cHRI. Abe et al.^79^ have suggested implementing robots capable of precise autonomous interactions with children.

The research questions have been organised as follows: Table 3 shows RQ1, RQ2, RQ4, RQ6, RQ7 and RQ8, while Figure 2 depicts RQ3, RQ5 and RQ7. The main purposes (RQ1) of the studies are disclosure of sensitive information^52^, social status estimation^75^, temperament estimation^76^, engagement measurement^77^, personality estimation^79^, and friendship estimation^80^. The age groups (RQ2) of children range from toddlers (21 months) to children up to 13 years old. The tasks (RQ3) in the interaction with the robots can be classified into three categories (see Figure 2): i) questionnaire or quizzes (2 studies^52, 75^), ii) open interactions (2 studies^76, 80^) and iii) activity-based tasks (2 studies^77, 79^). Regarding the robotic platform, Table 3 (RQ4) shows that only 1 study^76^ has not utilised a humanoid robot for experimentation, while all others have adopted a humanoid robot (e.g., Nao^52, 77^, LiPro^79^, Robovie^80^ and other humanoid robot^75^). As seen from the Figure 2, 1 study has an autonomous mode of operation (RQ5)^80^ while the rest are either semi-autonomous^75^, teleoperated^76, 79^ or operated using the WoZ setup^52, 77^. For the user sensing (RQ6), Table 3 shows that all the 6 studies have recorded data via audio-visual sensors (i.e., microphone and camera) of the robots and/or the environment. We identified that there are two types of analyses (RQ7) performed in these studies: statistical analysis (3 studies^52, 77, 80^) and machine learning-based analysis (3 studies^75, 76, 79^). The analysis includes standard statistical analyses (between conditions of the experiment^52, 77^, or correlation^80^) or classification using Support Vector Machines (SVM) or convolution neural networks^75, 76, 79^. In terms of studies’ results (RQ8), we have explored how the robotised measurements perform in assessment of the variable under consideration: (1) children were more open to sharing information about other classmates being bullied to a robot than a human interviewer^52^, (2) the proposed algorithm for social status estimation using position tracking with a robot demonstrated 71.4 % accuracy^75^, (3) the proposed algorithm obtained 85% accuracy for temperament estimation^76^, (4) cross cultural differences were observed in engagement levels^77^, (5) the proposed personality assessment algorithm was demonstrated successfully as compared with chance results^79^, and (6) the proposed algorithm estimated 5% of the friendships with 80% accuracy in^80^.

### 4.2 Protocol Related Limitations

**Figure 3:**
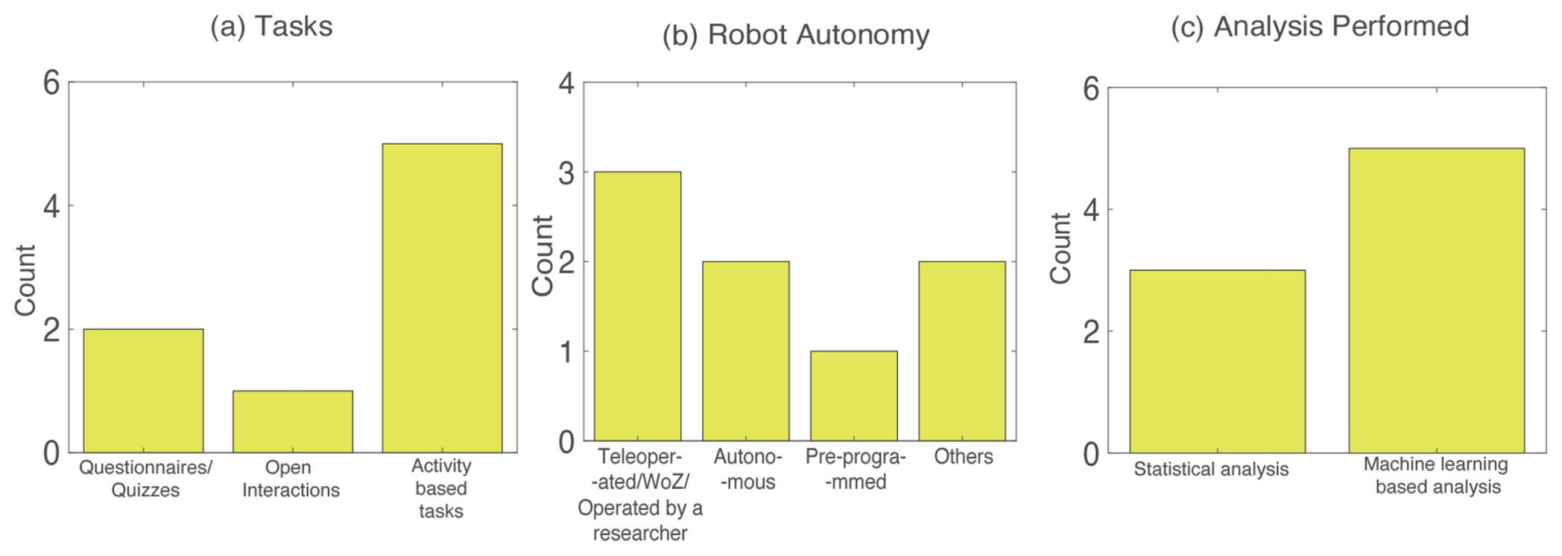
Bar graph showing the quantitative summary of the results for the ‘Protocol Related Limitations’ challenge: (a) Tasks performed in these studies (RQ3), (b) Robot autonomy in the studies screened (RQ5), and (c) Analysis performed in the above studies (RQ7).

Protocol related limitations include, but are not limited to, small sample sizes^78, 82^, participants with narrow age-range^75, 80^, testing out the technology with stakeholders^81^, better questionnaire/tasks during the interactions^52^, and biases in the annotation/scoring techniques^77, 79^ that could lead to inconsistencies in overall data analysis. Figure 3 and Table 4 show the results from studies that have mentioned ‘protocol related limitations’ as one of their challenges/future work. Our findings showed that 8 out of 10 of the screened studies highlighted protocol related limitations. The research questions have been organised as follows: Table 4 collects RQ1, RQ2, RQ4, RQ6, RQ7, and RQ8, and Figure 3 depicts RQ3, RQ5 and RQ7. The main purpose (RQ1) of the studies range from disclosure of sensitive information^52^, mood estimation^78^, personality estimation^79^, social status estimation^75^, engagement measurement^77^, friendship estimattion^80^, emotion estimation^81^ and mental state estimation^82^. The target user groups (RQ2) range from children of the ages of 3 to 13 years old. As seen in Figure 3, the experimental tasks (RQ3) include questionnaires in 2 studies^52, 75^, open/free interactions^80^ or activity based tasks in 5 studies such as dancing or imitation^78^, card-playing game^77, 82^, scenario-based task^81^ or a combination of games^79^ like playing dice, singing, hide and seek, foot race, playing rock-paper-scissors and many more. As seen in the Table 4, all studies have utilised humanoid robotic platforms (For example, Nao^52, 77^, LiPro^79^, RoBoHon^78^, Robovie^80^, DiGORO^82^ and other humanoid robot^75^) for conducting the study (RQ4) except^81^ which utilised both humanoid (Robotis OP2 and Robotis Mini) and non-humanoid (Romo) robots. Figure 3 shows that out of the 8 studies mentioned (RQ5), there are only three studies that follow an autonomous mode of robot operation^75, 78, 80^ while the rest are either pre-programmed^81^, semi-autonomous^82^, teleoperated or using the WoZ setup^52, 77, 79^. User sensing (RQ6) comprises primarily of audio-visual recording from robot-based and/or environment-based in all the studies that have been mentioned in Table 4. We also observe that the methodologies employed for data analysis (RQ7) predominantly range from standardised statistical analyses (between varying conditions of the experiment^52, 77^ or correlation based analysis^80^) for 3 studies or machine learning based analysis for the remaining 5 studies like probabilistic modelling^82^, reinforcement learning^78^ or classification using feature extraction followed by k-means clustering or SVM^75, 79, 81^ as observed in Table 4 and Figure 3. In terms of the studies results (RQ8), we have explored how the robotised measurements perform in investigating the variable under consideration: (1) children were more open to sharing information about other classmates being bullied to a robot than a human interviewer^52^, (2) the proposed personality assessment algorithm was demonstrated successfully as compared with chance results^79^, (3) the proposed method with the robot performed mood estimation and improvement^78^, (4) the proposed algorithm for social status estimation using position tracking with a robot demonstrated 71.4 % accuracy^75^, (5) cross-cultural differences in engagement levels have been observed^77^, (6) the proposed algorithm estimated 5% of the friendships with 80% accuracy in^80^,(7) the proposed algorithm performs real-time emotion classification with significant improvement in unweighted accuracies^81^, and (8) the proposed method of using a robotic playmate is accurate in mental state assessments^82^.

### 4.3 Establishing Less Constrained/More Naturalistic and Prolonged Interactions

**Figure 4:**
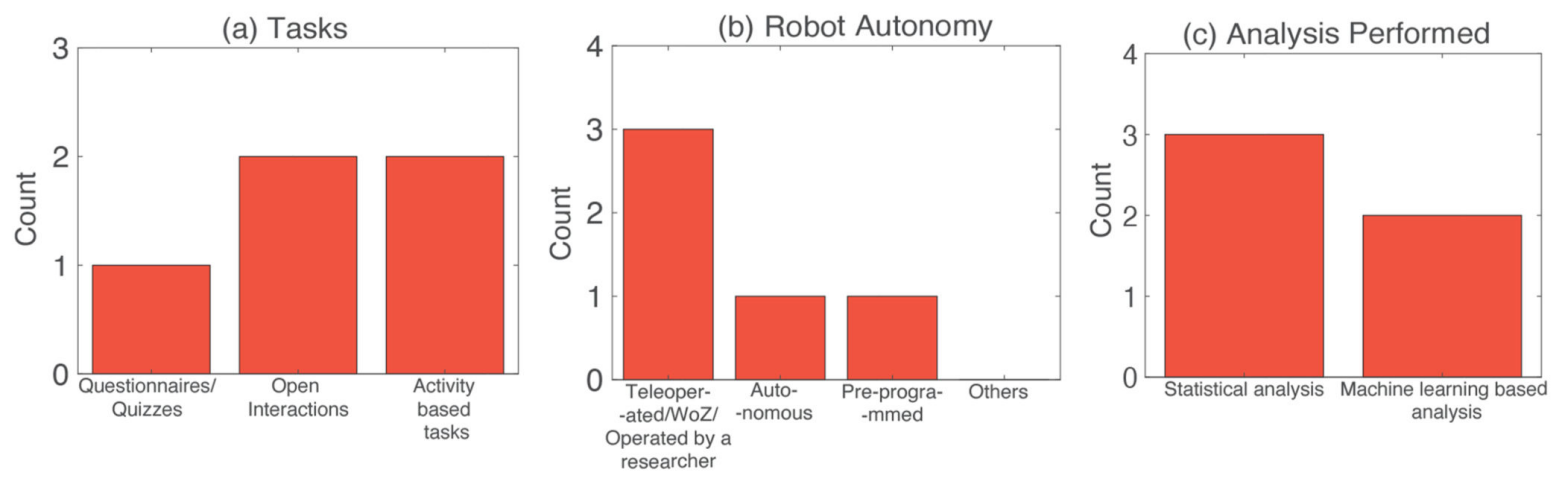
Bar graph showing the quantitative summary of the results for the ‘Establishing Less Constrained/More Naturalistic and Prolonged Interaction’ challenge: (a) Tasks performed in these studies (RQ3), (b) Robot autonomy in the studies screened (RQ5), and (c) Analysis performed in the above studies (RQ7).

Figure 4 and Table 5 show the results from studies that have mentioned ‘establishing less constrained/more naturalistic and prolonged interactions’ as one of their challenges/future works. Our findings showed that 5 out of the 10 studies discussed the challenges of running longitudinal, naturalistic and prolonged interaction to offer opportunities to conduct detailed and insightful studies. These include but are not limited to multi-session studies with less constrained interaction with the robot in order to promote relationship building. For example, Bethel et al.^52^ have suggested, as part of their future works, that longer and less constrained interactions might help in building child and robot relationships and might help in children to disclose sensitive information. Gamborino et al.^78^ have mentioned as part of their limitations/challenges, the investigation in the changes in accuracy of the proposed algorithm in determining the social profile of the children through the multi-session interactions. Sano et al.^76^ have suggested that their future works would entail time-series aspect of the child robot interaction. Rudovic et al.^77^ and Ismail et al.^83^ have both suggested conducting multi-session studies to improve the interaction experience for the participating children as part of their future works.

The research questions have been organised as follows: for RQ1, RQ2, RQ4, RQ6, RQ7 and RQ8, please refer to Table 5 while for RQ3, RQ5 and RQ7, please refer to the Figure 4. The purpose (RQ1) of the studies range from disclosure of sensitive information^52^, mood estimation^78^ as well as engagement^77^, temperament^76^ and concentration^83^ measurements (Table 5). The target user group (RQ2) range from toddlers (21 months) to 13 years of age. The tasks range (see Figure 4) from the psychological questionnaires based tasks^52^ (1 study), or activity based tasks (4 studies) like dancing, imitation games^78^, pairing cards^77^ and free interaction based tasks^76, 83^. Only 1 study has not used a humanoid robotic platform (Table 5 (RQ4))^76^, while the remaining (4 studies) have used a humanoid robot (e.g., Nao^52, 77, 83^ and RoBoHoN^78^). The autonomy of the robot predominantly is WoZ/teleoperated/operated by the researchers^52, 76, 77^, followed by autonomous^78^ and pre-programmed^83^, as depicted in Figure 4. All of the 5 studies have used audio-visual robot based sensing and/or environment based sensing (Table 5 (RQ6)). The analyses (Table 5 and Figure 4(RQ7)) were standardised signal processing and statistical analysis between different conditions of the experiment^52, 77, 83^ for 3 studies and classification pipeline through feature extraction, followed by convolutional neural networks for 1 study^76^ or reinforcement learning for 1 study^78^. In terms of the study results (RQ8), we have explored how the robotised measurements perform in investigating the variable under consideration: (1) children were more open to sharing information about other classmates being bullied to a robot than a human interviewer^52^, (2) the proposed method with the robot performed mood estimation and improvement^78^, (3)the proposed algorithm obtained 85% accuracy for temperament estimation^76^, (4) cross-cultural differences were observed in engagement levels^77^, and (5) increased eye contact was observed in robot-based intervention condition for ASD children as compared with the control condition of classroom interaction children^83^.

### 4.4 Development of a Validation Protocol

**Figure 5:**
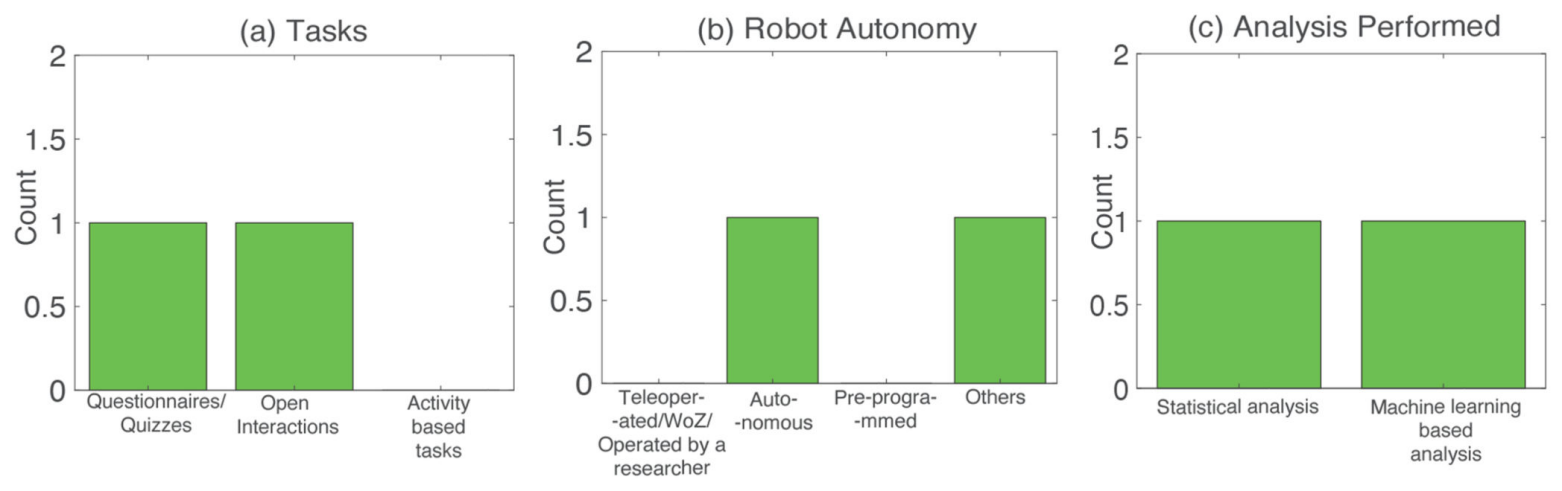
Bar graph showing the quantitative summary of the results for the ‘Development of a Validation Protocol’ challenge: (a) Tasks performed in these studies (RQ3), (b) Robot autonomy in the studies screened (RQ5), and (c) Analysis performed in the above studies (RQ7).

Figure 5 shows the results from studies that have mentioned ‘development of a validation protocol’ as one of their challenges/future works. Out of the 10 studies, only two studies^75, 80^ raises the challenge of the development of a validation protocol as shown in Figure 5. Komatsubara et al.^75^ claimed that while their proposed method has been effective in the measurement of social status, the validation of their computed evaluation needs to be performed by more precise protocols in order to generalise the results for varying population groups. Further, they mentioned that their follow up works would be in the domain of developing a baseline (comparing findings with the communication between children) in order to determine the efficacy of their proposed method. Kanda et al.^80^ have mentioned as part of their limitations, that a more generalisable friendship estimation algorithm can only be developed when their findings can be compared with observed user behaviour in the absence of a robot. To summarise, the main purpose (Table 6 (RQ1)) of the studies were social status estimation^75^ and friendship estimation^80^, while the target user group (Table 6 (RQ2)) of 5th grade children^75^ and healthy children between the age range of 11-12 years^80^. The task (Figure 5 (RQ3)) comprised of questionnaire/quiz based task^75^ (Figure 5) and open/free interactions^80^ while the robot platforms (Table 6 (RQ4)) employed were humanoid robots for both studies. The robot autonomy (Figure 5 (RQ5)) was semi-autonomous in^75^ and autonomous in^80^, while audio visual recording was performed using robot-based and environment-based cameras/sensors for user sensing (Table 6 (RQ6)) for both studies. The analysis (Figure 5 and Table 6 (RQ7)) consisted of standardised statistical analysis (correlation based)^80^ or machine learning pipeline of feature extraction (time spent alone and spent outside personal desk) followed by classification using SVM^75^. The main findings (Table 6 (RQ8)) of the studies include (1) the proposed algorithm for social status estimation using position tracking with a robot demonstrated 71.4 % accuracy in^75^, and (2) the proposed algorithm estimated 5% of the friendships with 80% accuracy in^80^.

## 5 Challenges in cHRI

Conducting cHRI studies could have important implications in developing socially intelligent technology, social and care robots that can be used for improvement of healthcare procedures. However, our results showed that cHRI studies are very challenging to conduct and certain limitations need to be addressed in order to conduct insightful studies especially in relation to mental wellbeing. Most cHRI studies are restricted by the limited functionality of the robotic platform^75–77, 79, 80^, as well as protocol related constraints such as small number of participants^78, 82^ and homogeneous sample pools, making their replicability across different user groups very challenging^84^. Another limitation of cHRI studies is that they are mainly short and one-off interactions^85^. While there are many studies that demonstrate the benefit of having a robot in the experimental setting, it is difficult to ascertain if the effect is long-lasting^85^. cHRI studies do not have a consistent ground truth (baseline so that the efficacy of the proposed robotised estimation can be measured^)75,80^; therefore, the validation of studies across other population samples becomes very difficult. Below we discuss the challenges that have been identified in the results section and how these limitations might be tackled in other cHRI studies or HRI studies that are not focusing on children but on other target user groups such as the elderly or adults.

### 5.1 Functionality of the robotised setup

Issues relating to the hardware of the robot platform or the limited sensing capabilities of the robot-based sensors posit a restriction while conducting experiments. Augmenting the robot functionality with additional sensing capabilities might enhance the cHRI experience by encouraging more precise measurements of the participants affect, engagement and cognitive abilities. For instance, in HRI, Alimardini et al.^86^ have used EEG signals to measure affect variations in their robot assisted mindfulness experiment sessions, and the accuracy of the robot interaction experience has been previously shown to be improved with the addition wearable sensors along with the traditional robot-based sensing^87^. Thus, addition of other user sensing techniques might enhance the limited capabilities of the robotised setup and enable more accurate measurements of user behaviour.

### 5.2 Protocol related limitations

With respect to small sample size, conducting studies on larger groups with varying demographics enables generalisability of the results. For instance, Rudovic et al.^77^ conducted robotised measurements across Serbia and Japan to investigate cross-cultural differences in engagement estimation. In HRI, Di Nuovo et al.^88^ have suggested to conduct clinical trials in order to determine the consistency of their proposed robotised assessments. In other cases, altering experimental protocol (introducing control conditions and annotations performed by multiple operators) might aid in conducting studies across a larger sample; thus enabling generalisabilty of the results across a non-homogeneous population pool.

However, conducting cHRI studies that can be generalisable across varying demographics has not been investigated in detail. Through collaborations, cHRI researchers might consider conducting studies across varying geographical locations, age groups, and gender, in order to make their research findings more generalisable and comprehensive.

### 5.3 Establishing longitudinal interactions

Often the cHRI studies are not prolonged and, thus, their effectiveness in the long term has not been explored in much detail. Moreover, in a psychological setting, longitudinal interactions are imperative, as building trust and rapport does not occur instantly^78^ . In the case of teleoperated experimental protocol, longitudinal studies become even more challenging as the experimental protocol might undergo inter-operator inconsistencies^89, 90^.

The ALIZ-E project has employed a ‘memory’ module in their cHRI setup where salient information from previous sessions were used to build up subsequent interactions, making each interaction more personalised/naturalistic than the previous one^91, 92^. Having prolonged interactions and using information from preceding interaction experience might account for the confounding aspect of the novelty effect that often influences the measurements, and might also encourage the development of more dynamic and adaptive robotic behaviours, making the interaction experience less constrained and more naturalistic. In the case of teleoperated experimental setup, ‘wizard training’ might help in maintaining consistent interactions over multiple sessions^90^.

### 5.4 Development of a validation protocol

It is very difficult to establish a consistent baseline (ground truth) to measure the effectiveness of the robotised assessments of user behaviour, especially as the cHRI studies have varying experimental protocols (autonomous or prescripted or WoZ). For instance, studies being conducted through the autonomous mode of operation might differ in evaluating the behaviour of the target population as well as their perception of the interaction experience as compared with other modes of robot autonomy such as Wizard of Oz^93^. Moreover, where the robot interaction occurs (in the lab or in the wild) might also contribute to the overall efficacy of measuring user behaviour^94, 95^. Studies have compared the diagnosis by the robot and the diagnosis by the subject matter experts^75^ in the field or other stakeholders, to determine the efficacy of the robotised measurements of the behaviour of the target population^96^. However, it is difficult to develop a ‘ground truth’ to measure the effectiveness of the robotised assessment that holds independently irrespective of the varying experimental protocols (mode of operation of robot platform and where the sessions are taking place).

### 5.5 Ethical concerns regarding using social robots for measurements in children

While the research involving social robots with children is becoming exceedingly prevalent, there are many ethical concerns that one should consider while using social robots especially for measurements in relation to mental wellbeing of children. Taking inspiration from Coeckelbergh et al.^97^, some important ethical issues that researchers must consider while designing cHRI studies with regards to mental wellbeing are:

1. Robot type: robot appearance (humanoid or vs non-humanoid), robot autonomy (autonomous, teleoperated, pre-scripted), behavioural capabilities (speech, gestures, postures).
2. Privacy matters: retrieving sensitive information from children and ensuring measures for safe storage and access of the collected information.
3. Attachments formed during interactions: in support of the assessment of mental wellbeing, should not cause distress when the robot has been removed.
4. Deception: robot’s capabilities, perception of robot’s behaviour.
5. Excessive reliance on technology: forming attachment with technology while retaining relationship building with other human beings.
6. Accountability and control: quality of the assessment performed, used as tools to aid the therapists and clinicians.

However, the ethical issues mentioned above^97^ have been investigated in relation to the responses of stakeholders and not children themselves and therefore should be considered only as a starting point for future research. cHRI researchers must ensure that the robot interaction in their respective studies conforms with the ethical standards in the field as well as the preference of the target population, as there can be serious implications for any shortcomings.

### 5.6 Conducting Multidisciplinary Research in cHRI

There is an increasing requirement to develop robots that are safe and conform with the cultural and behavioural norms of society. Just as robots need to understand the subtle behaviour manifestations of human behaviour, the end users should also understand the capabilities of the robot and what it can and cannot do^§^. This requires studying robotics from varying perspectives for successful integration of robots in everyday life. Thus, robotics studies should employ subject matter experts from the fields of computer science, psychology, medicine, and engineering^98^ . However, due to the varied strengths associated with the different fields, there are very often delays in the understanding and implementation of the final design of the robot behaviour, experiment setup, testing facilities and even recruitment of volunteers^99^. Applying the above mentioned principle to conducting cHRI research for mental wellbeing, we believe that it would require successful collaboration from subject matter experts as well as stakeholders (parents, teachers). Thus, cHRI researchers must understand the varying perspectives within their team-members as well as their end-users for seamless application of cHRI for assessing mental wellbeing in children.

## 6 Opportunities in cHRI Research for Mental Wellbeing

In this section, we have discussed some of the potential future avenues of cHRI research, especially in relation to mental wellbeing in children. Firstly, in the case of children, social robotics has been predominantly applied in the fields of autism research^38, 39^, edutainment^43^ and for providing companionship in medical scenarios^100^, thus, the application of cHRI for screening of mental wellbeing related concerns can be a promising avenue that researchers might like to consider. Secondly, even in the case of children as target user groups, their cognitive development is manifested in various behavioural and psychological changes in information processing, reasoning and reaction to various environmental stimuli^101–104^, thus, future research could focus on developing robotised wellbeing screening platform that takes into account the varying demographic factors (for example, age and gender). Thirdly, many studies^75, 76, 79^ also commonly employ various user and environment based sensing in order to augment their robotised setup, thus augmenting the existing capabilities of the robot might help future researchers in developing more realistic interaction experiences. Finally, due to the propagation of availability of resources in a remote/hybrid format during the pandemic, it is but natural to consider delivering robotised screening through other means that do not require physical presence in the lab; thus, constructing a remote robotised platform for wellbeing screening might be a potential avenue where cHRI research could be headed. These opportunities have been discussed in more detail below.

### 6.1 Using cHRI in Mental Wellbeing Screening

The use of child-robot interaction for psychological screening especially for detection of wellbeing related anomalies in healthy children has not been explored in much detail. Uchida et al.^96^ demonstrated that adults were more comfortable in disclosing negative information about themselves to a robot as compared with a human therapist. Moreover, Bethel et al.^13^ have demonstrated that preschool children were more reluctant to share a secret (information they were asked not to share in the experiment) with a human interviewer as compared with a humanoid interviewer. HRI research has already been used as a tool for improving healthcare related evaluation procedures. For example, Di Nuovo et al.^88^ have used robots to carry out the Montreal Cognitive Assessment (MOCA^¶^) test on the elderly using embedded sensors present on the robot and behavioural questionnaires for detecting cognitive impairment in the study participants. Moreover, robotic assisted practises have been employed to carry out medical examinations in geriatric care facilities using speech and tactile interfaces present on the robot^105^. In the case of children, robots have been used for delivering interview based evaluations in relation to issues concerning abuse, violence and bullying using speech and vision sensors present on the robot and the experiment room^14, 52^.

In the case of mental wellbeing related research, very often the markers of underlying psychological distress are not very apparent and are not brought to the surface until very late. Even children in regular contact with psychological services may find it difficult to confide in another person whom they might find intimidating^14, 20^. Robots might be helpful in identifying these cases, as research has suggested that children view robots as social peers and have shown to be more comfortable confiding in them^33, 106^. More recently, Abbasi et al.^107^ have reported that the robotised mode of conducting psychological questionnaires has been more successful in identifying cases with wellbeing related concerns as compared to standardised modes of self-report and parent-report test administration in children. Thus, building on this recent research, carrying out pro-active screening of wellbeing related anomalies in children using robotised measurement platforms might help in better management of mental health of the younger population.

### 6.2 Demographics relevant Mental Wellbeing Screening in Children

Many studies have shown that children of varying age groups exhibit marked differences in perception^108, 109^, intentional control over responses^110^ and knowledge and awareness of their surroundings^111, 112^. However, this significant factor has not been taken into account in the cHRI studies either at the study design stage or at the data processing stage.

Baxter et al.^45^ found significant differences in engagement levels (measured by gaze direction) in preschoolers during their first interaction with a social robot. They collected video data of children from two directions to compute the gaze patterns during their interaction with Nao. They found that younger children were more easily distracted and heavily relied on the human experimenters’ presence in the experiment room. Moreover, Sandygulova et al.^84^ reported that the perceived gender and age of the robot had differing effects on children belonging to varying age-groups and gender. Their results showed that children belonging to different age groups (8-12 years) and gender showed varying happiness levels (measured by the SHORE software through the real-time camera feed placed in front of the participant) while performing the experimental tasks. Further, Venture et al.^47^ have demonstrated that children belonging to the ages 3-5 years had different perception of the non-verbal communication exhibited by the robot (measured using two camera feeds from different viewpoints). The older children attributed significant meaning to the idleness of the robot while the younger children considered it as a toy and did not pay much attention to the non-verbal cues.

Due to varying cognitive levels of children belonging to different age and gender groups, developing a robotised wellbeing screening platform that accounts for this variation in knowledge, behaviour and responsiveness will be very beneficial in detection of anomalies in mental wellbeing.

### 6.3 User Sensing and cHRI Experience Evaluation

HRI studies commonly employ multimodal sensing to gain further insight into the interaction experience of the users. These include sensors embedded in the robot, sensors present on the users and/or sensors present in the environment^88, 105, 113^. These sensing technologies are being predominantly used for measuring user behaviour through tertiary metrics (e.g., participant’s responsiveness measured through gaze and/or amount of speech by robot based or environment based cameras). Building a framework that uses multimodal user sensing in addition to audio-visual sensing through robot or environment based sensors and behavioural questionnaires, might help in obtaining a precise measurement of the subject’s socio-emotional and cognitive responsiveness during their cHRI experience.

Using neurophysiological responses in addition to electrophysiological, audio-visual and behavioural information can provide a more accurate measurement of wellbeing in children. For example, in HRI, Alimardani et al.^86^ have used neurophysiological responses from the EEG signals collected during the experiment to measure affect changes during robot-assisted mindfulness sessions. The main findings are that EEG responses are promising in real-time detection and for providing neurofeedback of the user’s mindful state. However, the authors have also acknowledged that the neurophysiological changes may vary based on the meditation task and the tools used for recruitment. Moreover, Cross et al.^114^ investigated neurocognitive responses using Functional magnetic resonance imaging (fMRI) sessions conducted before and after a week of interaction with a social robot. They found that long-term interaction with the robot leads to an increase in empathy and emotional attachment with the social agent. Rauchbauer et al.^115, 116^ conducted fMRI sensing while the participants were conversing with a robot agent. Their findings suggest that interaction with a robot leads to increased neurocognitive responses from regions responsible for the executive and perceptual functioning of the brain. Kawaguchi et al.^117^ measured neurophysiological signals (Functional near-infrared spectroscopy, fNIRS) in children while they interacted with the PARO robot and found that reduction in cortical activity responsible for happy emotion processing of their brains were reduced after the interaction was over, suggesting enhancement of their mood.

Multimodal user sensing also gives insight into the cHRI experience from different aspects and can help in evaluating the full effect of the study experiment. For example, increase in brain activity in the emotion cortex while recalling memories^118^ might help in understanding the effectiveness of the robotised screening. Thus, conducting wellbeing analysis using standardised interviews might be augmented using a battery of data recorded from the sensors present on the robot, environment and the participants.

### 6.4 Remote Robotised Mental Wellbeing Screening

It is but natural to wonder about the feasibility of teletherapeutics especially in the domain of psychological screening. From a computer science perspective, affect studies have explored virtual and conversational agents for detection of variations in mood^23, 25^. From a psychology point of view, access to remote psychologists^119^ might help in disclosing more sensitive information since the participants are more comfortable in their own residences.

Studies have explored the use of virtual agents and conversational agents in intervention based studies like improvement of mood, anxiety, reducing stress, and in general promoting improved mental health^23, 25, 120^. Tsoi et al.^121^ designed VectorConnect, a robotic teleoperated system to combat social isolation in children. The interface allowed one child to control the robot placed in another child’s house, who was requested to give detailed instructions to the remote child for control and movement. Robotic sensors further allowed the remote child to perceive the residence environment, and the two children also communicated using video conferencing. Robinette et al.^120^ compared robotic instructions given in the robot’s virtual, remote, and actual presence. They observed that there was little difference between the three conditions in terms of instructions. Moreover, Li et al.^122^ reviewed about 33 experimental works that showed that while the users preferred the physical presence of the robot, there was no significant quantitative difference in the case of the remote presence of the robot and the virtual agent that were both presented on the camera feed. Remote robots (live or pre-recorded video of physically embodied robots) have a logistical advantage, compared with collocated robots, in terms of location and that user studies can be carried out without being physically present near the robot^120, 123^. The remote presence of the robot might also be beneficial in terms of information gathering, as all sensors on the robot could be potentially used even without collocation (in case of live interaction via video feed). Thus, exploring the possibility of remote psycho-therapeutics using remote robotised multimodal screening might be an exciting avenue of cHRI research for wellbeing evaluations.

## 7 Conclusions

In recent years, great advances have been made in cHRI research; however, using robots for mental wellbeing measurements is still an open challenge. In this paper, we present a narrative review of cHRI papers on the assessment of factors affecting mental wellbeing (social status, temperament, engagement, mood, personality, interaction with other humans, emotion, mental state and concentration) in children from the proceedings of IEEE ROMAN (2016-2021) and keyword-based article searching using Google scholar. We have used the SPIDER framework to search for relevant elements for the inclusion of short-listed studies. Our findings (10 screened papers in total) have identified several challenges: i) functionality of the robotised setup, ii) protocol related limitations, iii) establishing less constrained, more prolonged naturalistic interactions, and iv) development of a validation protocol. We have also categorised the current research in terms of robot-related factors (robot autonomy and type of robot), protocol-related factors (experiment purpose, tasks, participants and user sensing) and data-related factors (analysis and findings). These challenges offer potential opportunities for cHRI research, especially concerning conducting mental health-related evaluation procedures.

We understand that some relevant studies might have been excluded from this work due to unclear titles and keywords. While we have tried to follow structured selection criteria for the inclusion of studies, since this review is a narrative review, we acknowledge that there might be some selection bias. We also acknowledge that, while we have categorised the challenges that cHRI researchers have faced in the past into these four broad categories, researchers might face other challenges specific to their work. However, we hope that this work provides cHRI researchers with some helpful recommendations while designing their studies about mental wellbeing. The development of socially intelligent robots for carrying out mental wellbeing measurements for children can have a lasting positive impact on their mental health and, subsequently, on various aspects of their adult lives. We genuinely hope that the insights presented in this paper will contribute to developing effective and efficient robots to assist in this process.

## Figures and Tables

**Table 1:** Elements of SPIDER as applied in this work.

Elements of SPIDER	Application in this work
S- Sample	Children of all age groups (0-18 years)
PI- Phenomenon of Interest	Robot based assessments in relation to mental wellbeing
D- Design	Studies using qualitative and/or mixed research methods (with a qualitative component)
E- Evaluation	Challenges, limitations and future works of the identified studies
R- Research type	Empirical studies

**Table 2:** Screened studies have been categorised according to the challenges/future work mentioned by them: functionality of the robotised setup, protocol related limitations, establishing less constrained/more naturalistic and prolonged interactions and development of a validation protocol.

Study	Functionality of the robotised setup	Protocol related limitations	Establishing less constrained/more naturalistic and prolonged interactions	Development of a validation protocol
52	✓	✓	✓	
75	✓	✓		✓
76	✓		✓	
77	✓	✓	✓	
78		✓	✓	
79	✓	✓		
80	✓	✓		✓
81		✓		
82		✓		
83			✓	

**Table 3:** Table showing the qualitative summary of the results for the ‘Functionality of the Robotised Setup’ challenge: (RQ1) Purpose, (RQ2) Target user groups, (RQ4) Robot platform, (RQ6) User sensing, (RQ7) Analysis, and (RQ8) Results.

Study	Purpose (RO1)	Target user groups (RO2)	Robot platform (RO4)	User sensing (RO6)	Analysis (RO7)	Results (RO8)
52	Disclosing sensitive information about being bullied	Healthy children (8 to 12 years)	Nao	Questionnaire responses from the participants	Statistical analysis between interviews conducted by the robot and human interviewer	Children were more willing to disclose information about other classmates being bullied to a robot than a human.
75	Social status estimation	Healthy children (5th graders)	Humanoid Robot	Audio-visual recording using robot based and environment based cameras and depth recording using Kinect	Features like time spent alone, spent outside personal desk and no of people surrounding the robot followed by classification using SVM	Social status estimation using position tracking of children when interacting with a robot was performed with 71.4% accuracy
76	Temperament estimation	Healthy toddlers (21 months to 41 months)	ChiCaRo	Video data from the camera on the robot and the ceiling, demographic data like age and questionnaire data rated by caregiver	Six set of features corresponding to average distance, average near distance, near distance ratio (using MASK RCNN) neutral emotion, happy emotion eye contact ratio SVM was used for temperament estimation Explainable AI algorithm named Shapley additive explanations was used to find which features were critical for the estimation model	Proposed technique demonstrated over 85% accuracy in temperament estimation for the mean of all factors related to temperament
77	Engagement measurement	Children with Autism (3-13 years)	Nao	Visual information recorded using cameras	Engagement, affect dimensions (valence, arousal and face expressivity) were coded on the Likert and ordinal scales and analysed across the studies conducted in Serbia and Japan	Exploratory analysis reveals cross cultural differences in engagement levels between the two groups
79	Personality evaluation	Healthy Children Mean Age: 5 years 9 months	LiPRO	Audio visual recording and depth recording using Microsoft Kinect	Features like distance from the robot, time spent around the robot, gaze and smile ratio followed by classification using SVM	Personality estimation was performed successfully as compared with chance results
80	Friendship estimation	Healthy children (11-12years)	Robovie	Audio-visual information using cameras and microphones in the environment, robot based actuators and sensors	RFID tag used to identify children, questionnaire data using questionnaires and video information to check consistency of the interaction	5% of friendships were estimated with 80% accuracy and 15% were estimated with 50% accuracy

**Table 4:** Table showing the qualitative summary of the results for the ‘Protocol Related Limitations’ challenge: (RQ1) Purpose, (RQ2) Target user groups, (RQ4) Robot platform, (RQ6) User sensing, (RQ7) Analysis, and (RQ8) Results.

Study	Purpose (RQ1)	Target user groups (RQ2)	Robot platform (RQ4)	User sensing (RQ6)	Analysis (RQ7)	Results (RQ8)
**52**	Disclosing sensitive information about being bullied	Healthy children (8 to 12 years)	Nao	Questionnaire responses from the participants	Statistical analysis between interviews conducted by the robot and human interviewer	Children were more willing to disclose information about other classmates being bullied to a robot than a human
**79**	Personality evaluation	Healthy Children Mean Age: 5 years 9 months	LiPRO	Audio visual recording and depth recording using Microsoft Kinect	Features like distance from the robot, time spent around the robot, gaze and smile ratio followed by classification using SVM	Personality estimation was performed successfully as compared with chance results
**78**	Mood estimation and improvement	Healthy children (mean age) 10.7 years	RoBoHoN	Visual cues through facial expression and body posture	Interactive Reinforcement Learning (IRL) with additional inputs from the researchers	Mood estimation was performed using visual cues and the proposedlRL algorithm enabled the robot to learn the social profile of the users
**75**	Social status estimation	Healthy children (5th graders)	Humanoid Robot	Audio-visual recording using robot based and environment based cameras and depth recording using Kinect	Features like time spent alone, spent outside personal desk and no of people surrounding the robot followed by classification using SVM	Social status estimation using position tracking of children when interacting with a robot was performed with 71.4% accuracy
**77**	Engagement measurement	Children with Autism (3-13 years)	Nao	Visual information recorded using cameras	Engagement, affect dimensions (valence, arousal and face expressivity) were coded on the Likert and ordinal scales and analysed across the studies conducted in Serbia and Japan	Exploratory analysis reveals cross cultural differences in engagement levels between the two groups
**80**	Friendship estimation	Healthy children (11-12 years)	Robovie	Audio-visual information using cameras and microphones in the environment, robot based actuators and sensors	RFID tag used to identify children, questionnaire data using questionnaires and video information to check consistency of the interaction	5% of friendships were estimated with 80% accuracy and 15% were estimated with 50% accuracy
**81**	Emotion estimation	Children with autism	Robotis OP2, Robotis Mini and Romo	Audio and visual robot based sensing and training of the algorithm done using online datasets	Emotion classification algorithm was trained using the IEMOCAP database (linear kernel function in SVM) and speech signal during the experiment were extracted (PCA and then mean and variances) to further train the algorithm	Statistical significance between the proposed real-time emotion classification and previous method in the improvement of unweighted accuracies
**82**	Mental state estimation	Healthy children (M age: 5.5years)	DiGORO	Audio-visual robot based and environment based sensing	Regularity of gaze, smile intensities and motions have been used for estimation of mental state and transition between states was done using probabilistic modelling	Playmate robotic system capable of assessment of mental state and switching between tasks to maintain engagement

**Table 5:** Table showing the qualitative summary of the results for the ‘Establishing Less Constrained/More Naturalistic and Prolonged Interaction’ challenge: (RQ1) Purpose, (RQ2) Target user groups, (RQ4) Robot platform, (RQ6) User sensing, (RQ7) Analysis, and (RQ8) Results.

Study	Purpose (RQ1)	Target user groups (RQ2)	Robot platform (RQ4)	User sensing (RQ6)	Analysis (RQ7)	Results (RQ8)
52	Disclosing sensitive information about being bullied	Healthy children (8 to 12 years)	Nao	Questionnaire responses from the participants	Statistical analysis between interviews conducted by the robot and human interviewer	Children were more willing to disclose information about other classmates being bullied to a robot than a human.
78	Mood estimation and improvement	Healthy children (mean age) 10.7 years	RoBoHoN	Visual cues through facial expression and body posture	Interactive Reinforcement Learning (IRL) with additional inputs from the researchers	Mood estimation was performed using visual cues and the proposed IRL algorithm enabled the robot to learn the social profile of the users
76	Temperament estimation	Healthy toddlers (21 months to 41 months)	ChiCaRo	Video data from the camera on the robot and the ceiling, demographic data like age and questionnaire data rated by caregiver	Six set of features corresponding to average distance, average near distance, near distance ratio (using MASK RCNN) neutral emotion, happy emotion eye contact ratio SVM was used for temperament estimation Explainable AI algorithm named Shapley additive explanations was used to find which features were critical for the estimation model	Proposed technique demonstrated over 85% accuracy in temperament estimation for the mean of all factors related to temperament
77	Engagement measurement	Children with Autism (3-13 years)	Nao	Visual information recorded using cameras	Engagement, affect dimensions (valence, arousal and face expressivity) were coded on the Likert and ordinal scales and analysed across the studies conducted in Serbia and Japan	Exploratory analysis reveals cross cultural differences in engagement levels between the two groups
83	Concentration estimation	Children with Autism	Nao	Audio-visual information recorded using environment based cameras and one camera attached to the chest of the robot	Eye contact measurement done manually using a stop watch to determine concentration by observation of video feeds through a certified personnel	ASD children have more eye contact in the robot based intervention program condition as compared with the control condition of classroom interaction

**Table 6:** Table showing the qualitative summary of the results for the ‘Development of a Validation Protocol’ challenge: (RQ1) Purpose, (RQ2) Target user groups, (RQ4) Robot platform, (RQ6) User sensing, (RQ7) Analysis, and (RQ8) Results.

Study	Purpose (RO1)	Target user groups (RO2)	Robot platform (RO4)	User sensing (RO6)	Analysis (RO7)	Results (RO8)
75	Social status estimation	Healthy children (5th graders)	Humanoid Robot	Audio-visual recording using robot based and environment based cameras and depth recording using Kinect	Features like time spent alone, spent outside personal desk and no of people surrounding the robot followed by classification using SVM	Social status estimation using position tracking of children when interacting with a robot was performed with 71.4% accuracy
80	Friendship estimation	Healthy children (11-12 years)	Robovie	Audio-visual information using cameras and microphones in the environment, robot based actuators and sensors	RFID tag used to identify children, questionnaire data using questionnaires and video information to check consistency of the interaction	5% of friendships were estimated with 80% accuracy and 15% were estimated with 50% accuracy

## Footnotes

*
https://www.cdc.gov/childrensmentalhealth/data.html

†
https://www.softbankrobotics.com/emea/en/index

‡
https://www.softbankrobotics.com/emea/en/support/nao-6/part-my-robot-too-hot

§
https://www.science.org/doi/epdf/10.1126/scirobotics.abl7022

¶
https://www.mocatest.org/
